# Revisitando o Intervalo QT: Um Antigo Marcador para uma Nova Doença?

**DOI:** 10.36660/abc.20220878

**Published:** 2023-01-31

**Authors:** Luiz Eduardo Montenegro Camanho

**Affiliations:** 1 Hospital Pró-Cardíaco Rio de Janeiro RJ Brasil Hospital Pró-Cardíaco – Serviço de Arritmias e Estimulação Cardíaca Artificial, Rio de Janeiro, RJ – Brasil

**Keywords:** Intervalo QT, QT Longo, Síndrome QT Longo

A infecção pelo SARS-Cov-2 (Covid-19) teve seus primeiros casos relatados em dezembro de 2019 na China e rapidamente se alastrou pelo mundo, sendo anunciada como pandemia em março de 2020 pela Organização Mundial da Saúde. As manifestações respiratórias são as clássicas, porém, as complicações cardiovasculares podem ocorrer por um comprometimento cardíaco indireto ou por uma ação direta do vírus no tecido miocárdico. Como consequência podemos encontrar insuficiência cardíaca, miocardite, arritmias e choque cardiogênico.^1^

O intervalo QT, medido do início do complexo QRS até o final da onda T, representa o processo de despolarização e repolarização ventricular, e varia com a frequência cardíaca. Os valores normais do intervalo QT corrigido (QTc) seriam ≥ 450 ms em homens e ≥ 460 ms em mulheres. O prolongamento do intervalo QTc ≥ 500 ms está fortemente associado a ocorrência de arritmias malignas e morte súbita. A cada 10 ms de incremento do intervalo QTc há um aumento de 5 a 7% do risco de ocorrência de torsade de Pointes(TdP) e a cada 20 ms há um risco iminente substancial.^2^

A síndrome do QT longo adquirido está classicamente relacionada a prescrição de drogas e distúrbios eletrolíticos associados,^3^ além de diversos outros fatores clínicos e comorbidades, tais como, idade, sexo, cardiomiopatias em geral, pós-infarto do miocárdio, sangramento intracraniano, diabetes, hipogonadismo, doença pulmonar crônica, entre outras.^4 - 7^

No entanto, o prolongamento do intervalo QTc como fator de risco isolado e marcador de mortalidade a curto e longo prazo, já foi amplamente estudado e descrito em diversas situações clínicas.

Ko et al. demonstraram uma série de 408 pacientes consecutivos submetidos a transplante hepáticoem que pode se observar um prolongamento do QTc em 70,9% dos pacientes no período pré-transplante, com aumento expressivo no pós-operatório imediato em 20% dos indivíduos. Entretanto, estes achados não se correlacionaram com a taxa de complicações ou mortalidade a longo prazo, com resolução eletrocardiográfica na grande maioria dos casos. Os autores sugerem que o prolongamento do intervalo QTc seja uma característica eletrofisiológica da cardiomiopatia cirrótica e, a sua normalização, um componente reversível desta condição.^8 , 9^

A doença cardiovascular é principal causa de morbidade e mortalidade nos indivíduos portadores de insuficiência renal crônica, conforme uma recente revisão sobre o assunto. A despeito das diversas alterações encontradas no estágio final da doença renal e que contribuem para o surgimento do QT longo e subsequente risco de TdP, os autores sugerem que o prolongamento do QTc, nesta condição, é um fator de risco independente para morte súbita e mortalidade por todas as causas.^10^

Hohneck et al. analisaram 105 pacientes com síndrome de Takotsubo que foram divididos em dois grupos: QT longo (69,5%) e QT normal (30,5%), seguidos por um tempo médio de 4,2 anos. A ocorrência de arritmias malignas durante os 30 dias iniciais foi similar em ambos os grupos (10,9% vs. 12,5%), mas a partir da análise multivariada, os autores concluem que o prolongamento do intervalo QTc na admissão hospitalar é um preditor negativo independente para desfechos desfavoráveis associados a esta condição.^11^

Em um elegante estudo, Gibbs et al. descreveram a mortalidade entre 30 dias e 3 anos de 980 pacientes internados em um hospital geral com QTc ≥500 ms comparado com 980 pacientes com intervalo QTc < 500 ms, por causas clínicas diversas e já ajustado para idade, sexo, index Charlson, admissão prévia e diagnóstico principal. Os autores concluem que o QTc ≥ 500 ms é um importante preditor de mortalidade a curto prazo e, que a longo prazo, as comorbidades encontradas sejam o fator principal relacionado a taxa de mortalidade geral.^12^

No cenário da infecção por Covid-19, o prolongamento do intervalo QTc já foi amplamente apresentado. Dentre os fatores clínicos mais importantes e associados estão a idade mais avançada, sexo feminino, distúrbios eletrolíticos e interações farmacológicas. Vale ressaltar que a polifarmácia é um item importante neste cenário clínico e deve ser sempre uma preocupação pelos riscos iminentes de TdP e morte súbita associada.^13 , 14^

Nesta revista, Barbosa et al. apresentaram os resultados de um estudo observacional que envolveu uma expressiva coorte retrospectiva de 1.296 pacientes admitidos em hospital terciário com o diagnóstico de infecção por SARS-Cov-2 no período de março de 2020 a 31 de julho de 2021.^15^Destes, 127 (9,8%) apresentaram intervalo QTc prolongado. A mortalidade e o tempo de permanência hospitalar forammaiores neste grupo quando comparados aos indivíduos com QTc normal. Na análise multivariada, houve associação significativa entre mortalidade e prolongamento do intervalo QTc, após controle para idade, sexo masculino, doença renal e índice de comorbidade de Charlson >3. As comorbidades associadas ao aumento da mortalidade foram: hipertensão arterial sistêmica, doença renal, DPOC e Charlson >3.

Os autores concluíram então que a mortalidade hospitalar por SARS-Cov-2 está associada ao prolongamento do intervalo QTC na admissão.

O desenho da pesquisa e a proposta dos autores não são inéditos, muito pelo contrário. Já há muitos anos o prolongamento do intervalo QTc vem sendo estudado como um marcador de risco cardiovascular a curto e longo prazo. Entretanto, o presente estudo traz informação relevante no cenário de uma nova doença que se apresenta com tantos desafios clínicos. A despeito da tão esperada redução nos casos graves de Covid-19 observada nos últimos meses, os dados apresentados nos fazem refletir sobre a necessidade de revisitar um velho e simples marcador (intervalo QTc), que nos auxilie a identificar subgrupos de pior evolução em uma doença potencialmente grave. Marcadores de gravidade são sempre bem-vindos na prática clínica.
