# Supplementary material for: Revisitando o Intervalo QT: Um Antigo Marcador para uma Nova Doença?
Source: Arq Bras Cardiol. 2023 Jan 31;120(1):e20220878. [Article in Portuguese] doi: 10.36660/abc.20220878 (PMC10389110; doi:10.36660/abc.20220878)
Supplement: Translation

# Translation: Revisiting the QT Interval: An Old Marker for a New Disease?

**Authors:** Luiz Eduardo Montenegro Camanho

**Affiliations:** 1 Brazil Hospital Pró-Cardíaco – Serviço de Arritmias e Estimulação Cardíaca Artificial, Rio de Janeiro, RJ – Brazil

**Keywords:** QT interval, Long QT, Long QT Syndrome

The SARS-Cov-2 (Covid-19) infection had its first cases reported in December 2019 in China and quickly spread worldwide, being announced as a pandemic in March 2020 by the World Health Organization. Respiratory manifestations are classic; however, cardiovascular complications can occur due to an indirect cardiac involvement or a direct action of the virus in the myocardial tissue. Consequently, we can find heart failure, myocarditis, arrhythmias, and cardiogenic shock.^1^

The QT interval, measured from the beginning of the QRS complex to the end of the T wave, represents the process of ventricular depolarization and repolarization and varies with heart rate. Normal values for the corrected QT interval (QTc) would be≥450 ms in men and≥460 ms in women. Prolongation of the QTc interval≥500 ms is strongly associated with the occurrence of malignant arrhythmias and sudden death. Every 10 ms of QTc interval increment, there is a 5 to 7% increase in the risk of torsade de Pointes (TdP); every 20 ms, there is a substantial imminent risk.^2^

Acquired long QT syndrome is classically related to prescription drugs and electrolyte disturbances associated,^3^in addition to several other clinical factors and comorbidities, such as age, gender, cardiomyopathies in general, post-myocardial infarction, intracranial bleeding, diabetes, hypogonadism, chronic lung disease, among others.^4 - 7^

However, QTc interval prolongation as an isolated risk factor and marker of short- and long-term mortality has already been widely studied and described in several clinical situations.

Ko et al. demonstrated a series of 408 consecutive patients undergoing liver transplantation in which QTc prolongation was observed in 70.9% of patients in the pre-transplantation period, with a significant increase in the immediate postoperative period in 20% of individuals. However, these findings did not correlate with complications or long-term mortality rate, with electrocardiographic resolution in the vast majority of cases. The authors suggest that prolonging the QTc interval is an electrophysiological characteristic of cirrhotic cardiomyopathy, and its normalization is a reversible component of this condition.^8 , 9^

According to a recent review, cardiovascular disease is the main cause of morbidity and mortality in individuals with chronic renal failure. Despite the various alterations found in end-stage renal disease that contribute to the onset of long QT and subsequent risk of TdP, the authors suggest that QTc prolongation, in this condition, is an independent risk factor for sudden death and mortality for all causes.^10^

Hohneck et al. analyzed 105 patients with Takotsubo syndrome divided into two groups: long QT (69.5%) and normal QT (30.5%), followed for a mean time of 4.2 years. The occurrence of malignant arrhythmias during the initial 30 days was similar in both groups (10.9% vs. 12.5%). However, from the multivariate analysis, the authors conclude that QTc interval prolongation on hospital admission is an independent negative predictor for unfavorable outcomes associated with this condition.^11^

In an elegant study, Gibbs et al. described the 30-day to 3-year mortality of 980 patients admitted to a general hospital with QTc≥500 ms compared to 980 patients with QTc interval<500 ms, due to different clinical causes and already adjusted for age, sex, Charlson index, previous admission, and main diagnosis. The authors conclude that the QTc≥500 ms is an important predictor of mortality in the short term and, in the long term, the comorbidities found are the main factor related to the overall mortality rate.^12^

In the scenario of Covid-19 infection, QTc interval prolongation has already been widely presented. Among the most important and associated clinical factors are older age, female gender, electrolyte disturbances, and pharmacological interactions. It is noteworthy that polypharmacy is an important item in this clinical scenario and should always be a concern due to the imminent risks of TdP and associated sudden death.^13 , 14^

In this journal, Barbosa et al. presented the results of an observational study that involved a significant retrospective cohort of 1,296 patients admitted to a tertiary hospital with a diagnosis of SARS-Cov-2 infection from March 2020 to July 31, 2021.^15^Of these, 127 (9, 8%) had a prolonged QTc interval. Mortality and length of hospital stay were higher in this group compared to individuals with normal QTc. In the multivariate analysis, there was a significant association between mortality and QTc interval prolongation after controlling for age, male gender, renal disease, and Charlson comorbidity index>3. Comorbidities associated with increased mortality were: systemic arterial hypertension, kidney disease, COPD, and Charlson>3.

The authors then concluded that in-hospital mortality from SARS-Cov-2 is associated with prolongation of the QTC interval on admission.

The research design and the authors’ proposal are not unprecedented, quite the contrary. QTc interval prolongation has been studied for many years as a marker of cardiovascular risk in the short and long term. However, the present study brings relevant information in the context of a new disease that presents many clinical challenges. Despite the long-awaited reduction in severe cases of Covid-19 observed in recent months, the data presented make us reflect on the need to revisit an old and simple marker (QTc interval), which helps us to identify subgroups with worse evolution in a potentially serious illness. Gravity markers are always welcome in clinical practice.
